# 

**Published:** 1867-01

**Authors:** 


### CONTENTS.

ORIGINAL COMMUNICATIONS.

Alveolar Abscess.... 1
Dental Caries, Hygienically Considered.... 13
Inquiry and Answer.... 17
An Address.... 21
Dental Nomenclature.... 25
The Use of the File in Dentistry.... 26
Cracking of Rubber Plates.... 38
Violence in Dental Operations.... 39

PROCEEDINGS OF SOCIETIES.

Association of the Colleges of Dentistry.... 29
Dental Association of Canada West.... 33
Delaware Dental Association.... 36
American Dental Convention.... 36
Notice.... 37

EDITORIAL.

Redman's Mallet Plugger.... 40
Delay.... 41
Personal—Prof. Watt.... 41
Nip and Snarler.... 41
Meetings.... 42
A New Extension Bracket.... 43
Notice.... 43
Errata.... 44

